# Investigating the effect of dielectric barrier discharge cold plasma on the aflatoxin removal and the physicochemical properties of sesame seeds

**DOI:** 10.1038/s41598-025-17731-6

**Published:** 2025-09-01

**Authors:** Asma Ghaani, Moein Bashiry, Parvin Zohrabi, Vahid Siahpoush, Neda Mollakhalili Meybodi, Mandana Dousti, Khadije Abdolmaleki

**Affiliations:** 1 Student Research Committee, Department of Food Science and Technology, School of Nutrition Sciences and Food Technology, Kermanshah University of Medical Sciences, Kermanshah, Iran; 2 Nutrition Sciences and Food Technology Research Center, Health Institute, Kermanshah University of Medical Sciences, Kermanshah, Iran; 3 Research Center for Environmental Determinants of Health (RCEDH), Health Institute, Kermanshah University of Medical Sciences, Kermanshah, Iran; 4 Department of Plasma Physics and Technology, Faculty of Physics, University of Tabriz, Tabriz, Iran; 5 Department of Food Sciences and Technology, School of Public Health, Shahid Sadoughi University of Medical Sciences, Yazd, Iran; 6 Research Center for Food Hygiene and Safety, Shahid Sadoughi University of Medical Sciences, Yazd, Iran; 7 Research Center of Oils and Fats, Kermanshah University of Medical Sciences, Kermanshah, Iran

**Keywords:** DBD cold plasma, Mycotoxin, Sesame seed, Textural properties, Aflatoxin, Peroxide value, Physicochemical properties, Biophysics, Diseases, Health care, Chemistry, Engineering

## Abstract

Cold plasma is a non-thermal technology that has been proposed as an efficient method for the mycotoxins destruction in the food industry. The first stage of this study was conducted to determine the effect of the dielectric barrier discharge (DBD) plasma treatment gas (air, argon, wet argon) on the aflatoxin B_1_ reduction and sesame seed peroxide value (PV). The results showed that air gas was more effective in the reduction of aflatoxin B_1_ from 65 to 47 ppb (27.7% decrease), while the peroxide content increased from 0.61 ± 0.09 to 1.61 ± 0.011. The effects of power (45 and 56 W) and exposure time (1, 4, and 8 min) of plasma with argon gas on aflatoxin B_1_ content and physicochemical characteristics of sesame seeds were studied in the second stage. The greatest reduction in aflatoxin B_1_ content was achieved at DBD treatment of 8 min and 56 W from 63.4 to 19.9 ppb (68.59% decrease). This sample was further investigated for physicochemical analysis in which PV, acid value, colour parameters (a* and b*), and texture parameters (hardness, fracturability, cohesiveness, gumminess) in the treated sample has been increased, whereas the pH, colour parameter (L*), texture parameter (springiness index), phytosterol, sesamol, sesamin and sesamolin levels has been decreased compared with the control samples. Also, there was no significant difference in protein content. The results indicate the effectiveness of DBD plasma treatment on AFB_1_ reduction, along with physicochemical changes that need to be optimized.

## Introduction

Mycotoxins are a group of toxic chemical compounds and stable secondary metabolites with low molecular weight that are naturally produced by certain types of fungi. The fungi that produce these toxic compounds include *Fusarium*, *Penicillium*, *Aspergillus*, *Alternaria*, and *Claviceps*. Mycotoxins may cause acute or chronic problems such as carcinogenesis, mutagenicity, teratogenicity, hepatotoxicity, immunosuppression, and endocrine disruption, depending on the type and metabolic conditions in the body, age, nutrition, and exposure duration^1^. These mycotoxins can be produced in different types of food, including seeds, fruits, vegetables, and nuts, and they threaten the health of consumers^2^. Convincing conditions for the growth of mycotoxins include high humidity, moderate temperature, hard weather conditions, vector insects, and food damage during improper storage and processing^3^. Nowadays, more than 500 types of mycotoxins have been identified, and aflatoxins (AFs), deoxynivalenol (DON), ochratoxin A (OTA), zearalenone (ZEN), patulin (PAT), fumonisin (FUM), and citrinin (CTN) are the most common types of mycotoxins that have been considered.

Aflatoxins are known as potential carcinogens in the mycotoxin group^4^. Aflatoxins are divided into six different types, Aflatoxins-B_1_ (AFB_1_), Aflatoxins-B_2_ (AFB_2_), Aflatoxin G_1_ (AFG_1_), Aflatoxins G_2_ (AFG_2_), Aflatoxin M_1_ (AFM_1_), and Aflatoxin M_2_ (AFM_2_). Out of these B_1_, B_2_, G_1_, and G_2_ are found in food crops or their products, while M_1_ (metabolite B_1_) and M_2_ are found in the animals’ by-products, such as dairy products. Aflatoxins B_1_, B_2_, G_1_, and G_2_ which are mainly produced by *Aspergillus flavus* and *Aspergillus parasiticus*. Aflatoxins B_1_ and B_2_ have a difuro-coumaro-cyclopentenone structure and emit blue fluorescence, whereas Aflatoxins G_1_ and G_2_ have hexagonal lactone rings that emit yellow-green fluorescence. Aflatoxins B_1_ and G_1_ have a double bond at the C8-C9 position, whereas aflatoxins B_2_ and G_2_ do not have this bond and are less toxic than B_1_ and G_1_^5^. Among all aflatoxins, aflatoxin B_1_ is known as the strongest carcinogenic agent, especially for liver cancer^2^. Therefore, the International Agency for Research on Cancer (IARC) has classified these types of aflatoxins as Group 1 carcinogens^6^. The European Union is very strict on aflatoxin B_1_, and according to the EU directive, the maximum level of aflatoxin B_1_ in oilseeds and cereals should not exceed 2 µg/kg^7^. Aflatoxin B_1_ is abundant in various products such as cereals, oilseeds, spices, and nuts.

Sesame seeds contain oil (44%-58%), protein (16%-28%), carbohydrates (13.5%), and ash (5%), and are high in minerals such as calcium, phosphate, iron, potassium, and vitamins for instance E, thiamin and niacin, as well as lignins, and phytosterols. These oilseeds have antioxidant, anti-inflammatory, antifungal, antiviral, and antibacterial properties. Sesame seeds are widely used in bakery products, pastries, and traditional desserts such as halva, tahini, and sesame butter^8^. In case of improper storage of sesame seeds, cellular respiration is still active, as a result of which the temperature and humidity of the storage environment increase and cause fungal toxins, especially aflatoxins, to be observed on the surface of sesame seeds^9^.

Various preservation methods are used to improve food quality and safety, including freezing, cooling, Pasteurization, and canning^10^. However, the use of non-thermal technologies, such as microwaves, ultrasound, pulsed light, gamma rays, and cold plasma, is preferred because they have less adverse effects on the physicochemical properties of food. Cold plasma technology (CP) is a new non-thermal technology used in the food industry. This technology has attracted considerable attention because it can be used to disinfect and sterilize, increase shelf life, reduce spoilage, and inactivate enzymes and microorganisms in food^11^. In the field of extraction, cold plasma has been used to improve the recovery of bioactive compounds such as phenols, essential oils, and antioxidants from plant matrices by increasing permeability and cell wall degradation^12^. Also, in the field of material modification, this technology has played an important role in improving the functional properties of cereal and legume proteins, modifying surface hydrophobicity, and improving the properties of packaging films^13^. Cold plasma is known as the fourth state of matter and partially ionized gas consisting of atoms, molecules, positive and negative ions, and reactive species of free radicals. Reactive oxygen species (ROS) and reactive nitrogen species (RNS) have been identified as the most active plasma species, and these species destroy organic compounds or inactivate microorganisms. Because of their high reactivity, ROS and RNS can react with all cellular components, resulting in the perforation and dispersion of macromolecules in cells, as well as inactivating pathogenic fungi and helping to stop the growth of mycotoxins^14^. Therefore, cold plasma technology is used to break down mycotoxins and convert toxic compounds into less toxic or non-toxic compounds. Various foods such as flour, wheat and barley, peanuts, dairy, and poultry products were exposed to cold plasma for microbial and mycotoxin decontamination^15^. For example, Feizollahi et al. (2020) investigated the effect of atmospheric pressure cold plasma on the deoxynivalenol degradation, quality parameters, and germination of barley grains^16^. Also, Dousti et al. (2024) studied the effect of dielectric barrier discharge (DBD) cold plasma treatment on the reduction of aflatoxin B_1_ and the physicochemical properties of oat^4^. The effect of dielectric barrier discharge high voltage atmospheric cold plasma on *Aspergillus flavus* inactivation and aflatoxin B_1_ degradation in inoculated raw peanuts was investigated^17^. However, little information is available on the use of DBD for AFB_1_ degradation and its effect on the physicochemical properties of sesame seeds under different conditions. Considering the nutritional value of sesame seeds, and the increasing use of its derived products, this study was conducted to investigate the effect of cold plasma treatment with different gases, exposure times, and powers on the reduction of aflatoxin B_1_ and the physicochemical characteristics of sesame seeds.

## Materials and methods

### Materials

AFB_1_ was purchased from Sigma-Aldrich (USA). Water, methanol, acetonitrile, chloroform, NaOH, n-hexane, and HCL (all HPLC grade) were obtained from Merck (Germany). A CA (cellulose acetate) syringe filter with a pore size of 0.22 μm was obtained from Microlab Scientific. Sesame seeds were procured from the local market of Kermanshah. All chemicals and reagents were of analytical grade.

### Dielectric barrier discharges cold plasma chamber (DBD-CP)

**Fig. 1:**
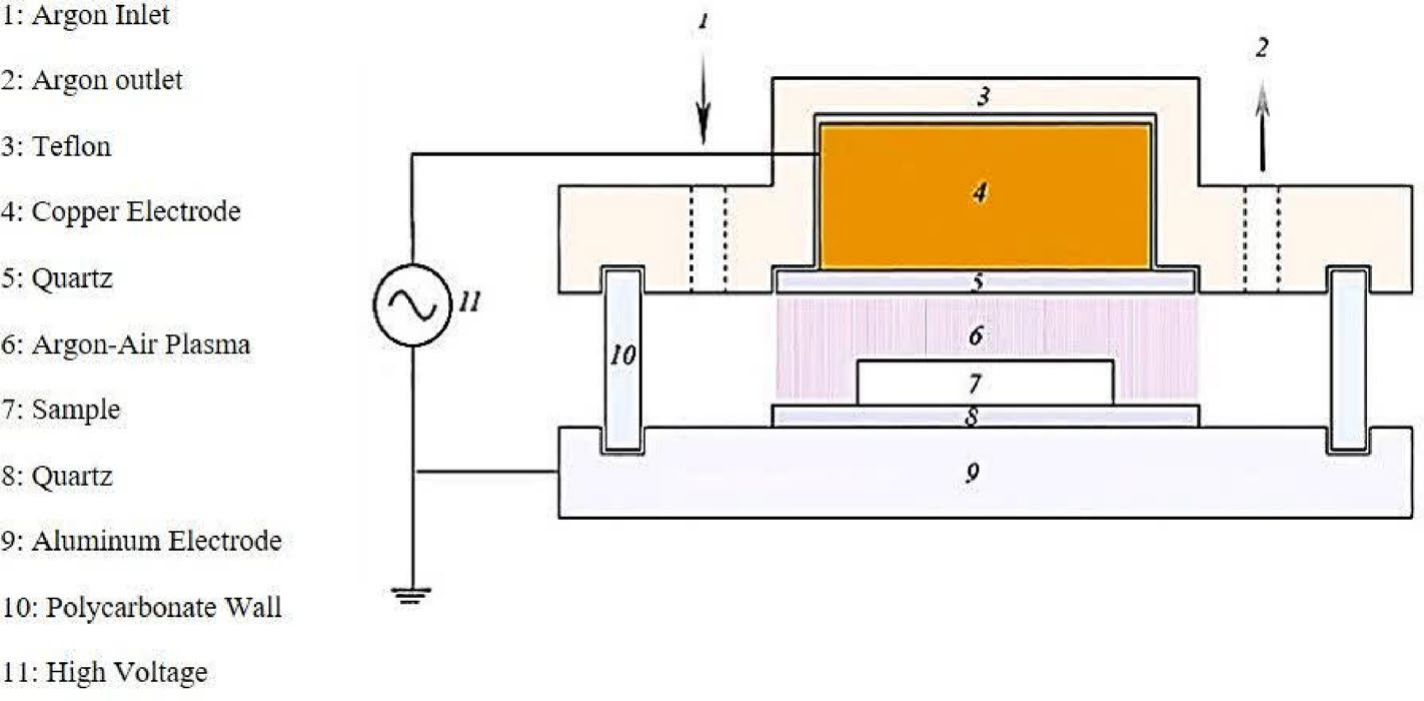
Schematic image of the plasma reactor.

In this study, a cylindrical dielectric barrier discharge (DBD) plasma chamber constructed by the Department of Physics at Tabriz University was used to generate cold plasma at atmospheric pressure. A schematic diagram of the reactor is shown in Fig. 1. In this reactor, a copper cylinder and a circular aluminum plate were used, both of which were covered by a 2-mm-thick quartz glass (dielectric barrier discharge) and acted as high-voltage and ground electrodes, respectively. Both the electrode and samples were placed in a cylindrical container made of transparent polycarbonate. Three different gas conditions, air (25% ambient humidity), wet argon (75% humidity), and argon (25% humidity), were introduced into the ambient air in the chamber as carrier gases, and the flow rate of the carrier gases was controlled by a flow meter. A sinusoidal AC voltage of 1–10 kV and a frequency of 35 kHz was applied to the electrodes. Stable and uniform microdischarges were observed in the mixture of all three types of carrier gas and the remaining background air between the electrodes at a distance of 5 mm, while all three types of gases were fed with a flow of 1 Nl/min. In addition, the first stage of the study was performed with a power of 40 W and at a time of 1 min. The second stage was also done with powers (45 and 56 W) and treatment times (1, 4, 8 min). All samples were stored in sterile, sealed polyethylene containers at 4 °C in the dark or dim light conditions for 24 h before aflatoxin measurement.

### Preparation of AFB_1_ solutions

A stock solution of AFB_1_ was prepared by dissolving 1 mg of AFB_1_ in 10 mL of HPLC-grade methanol. It was then stored in a dark glass vial at 20 °C. Standard working solutions at concentrations of 0.1, 0.5, 1, 5, 10, 20, 50, and 100 µg/L for calibration curves were prepared by adding a certain volume of the existing solution to methanol, which was maintained until injection into the HPLC at 4 °C.

### Determination of aflatoxin B_1_

In this study, the AZURA HPLC device from KNAVER was used to evaluate AFB_1_ concentration. The detection of aflatoxin was performed using a fluorescence detector (RF-20 A) at an excitation wavelength of 329 nm and an emission wavelength of 460 nm. The HPLC column used was a C18 column (5 μm, 4.6 mm x 250 mm). The column temperature was set to 40 °C. The mobile phase containing a mixture of acetonitrile and water was used at a volume ratio of 10:90 and a flow rate of 1.5 ml/min. The volume injected into the device was 20 µl.

### Physicochemical properties

To determine the effect of cold plasma treatment on the physicochemical properties of sesame seeds, the plasma treatment gas, which had the greatest effect on the reduction of aflatoxin B_1_ and increased the PV i.e., air gas, was used to treat the samples in the next phase of which following different physicochemical properties were studied.

#### Peroxide value (PV)

Before the peroxide test, sesame oil was extracted using a Soxhlet apparatus (Behr E4, Germany). Approximately 3 g of the oil sample was dissolved in 30 mL of glacial acetic acid and 20 mL of chloroform (volume ratio 3:2). Then, 1 mL of saturated potassium iodide solution was added. The solution was kept in the dark for 1 min. After adding 50 ml of distilled water and 1 ml of 1% starch, the solution was titrated with sodium thiosulfate (0.01 N) until the blue color disappeared. The amount of peroxide was calculated using the following formula in milliequivalents of oxygen/kg of oil^18^.

$$PV{\text{ }}value{\text{ }} = {\text{ }}1000{\text{ }}\left( {S{\text{ }} \times {\text{ }}N} \right){\text{ }}/W$$

Where S is the volume of sodium thiosulfate solution used in mL, N is the normality of sodium thiosulfate solution, and W is the weight of the oil sample (grams).

#### Acid value (AV)

To determine the acid value, sesame oil was extracted using a Soxhlet apparatus (Behr E4, Germany). Approximately 2 g of the oil sample was weighed in an Erlenmeyer flask.

Then, it was dissolved in 50 ml of a 1:1 mixture of benzene and ethanol and then titrated with 0.1 N ethanolic solution of potassium hydroxide (KOH) in the presence of 0.5% phenolphthalein (prepared in ethanol). The endpoint was indicated by a pink color for 30 s. A control titration was also performed with 50 ml of solvent; the acid value was calculated using the following equation:^19^.

$${\text{Acid value }} = ~\frac{{56.1\left( {{\text{A}} - {\text{B}}} \right) \times {\text{N}}}}{{\text{W}}}$$

Where 56.1 is the molar mass of potassium hydroxide (KOH) in grams per mole, A is the volume of KOH required for the titration of the sample, and B is the volume of KOH.

required to titrate the control, N is the normality of KOH, and W is the weight of the oil sample (grams).

#### pH value

The sesame seed samples were mixed with distilled water at a ratio of 1:10 and then homogenized. Finally, the pH of the samples was measured using a pH meter at ambient temperature.

#### Color analysis

The color measurement of sesame seed samples treated with cold plasma was performed using a HunterLab colorimeter (Konica Minolta Chroma meter CR-410, Japan). L* (lightness/darkness), a* (redness/greenness), and b* (yellowness/blueness) parameters were measured and reported^20^.

#### Determination of protein content

Protein was measured using the Kjeldahl method. First, 1 g of sample was placed in a Kjeldahl flask, and 5 g of catalyst containing 0.5 g of copper sulfate, 4.5 g of potassium sulfate, and 15 mL of 98% sulfuric acid were added. Then, the samples were placed on a heater for 2–3.5 h at a temperature of 300 °C to digest and become completely transparent, and after cooling, they were diluted with distilled water to 75 ml. Afterward, this solution was placed in the distillation system (Tecator Kjeltec 1030 Auto Analyzer), and finally, titration was performed. The amount of nitrogen was determined using the following formula, and the crude protein percentage was calculated by multiplying the total nitrogen by a factor of 6.25 ^21^.

Nitrogen (%)=(14×Acid normality× volume of acid used)/( sample weight gram×1000)×100.

#### Texture profile analysis (TPA)

Sesame seed texture parameters were calculated using a texture profile analyzer (TA20, KOOPA). The sample was placed into the plate and compressed to 50% of its original height at a speed of 0.1 mm/s using a 43-mm cylinder probe and a 5-kg load cell. The parameters of hardness, cohesiveness, fructibility, springiness, and gumminess were calculated using the texture profile analysis curves^22^.

#### HPLC analysis for phytosterols

HPLC (Agilent^®^, Santa Clara, CA, USA) with a quadruple pump was used for chromatographic separation. The samples were injected into column C18 (Phenomenex^®^, Torrance, CA, USA) at a volume of 20 µl. Separation was performed using isocratic elution with acetonitrile: methanol (70:30). The flow rate was 1 ml/min with the column temperature set at 35 °C. Sterols were identified using a variable wavelength detector (VWD) at 205 nm. Phytosterols were identified by comparison with valid standard retention times. Sterol content was measured as mg/kg of oil^23^.

#### HPLC analysis for sesamol, sesamin, and Sesamolin

Sesamol, sesamin, and sesamolin were analyzed using an Alliance model 2695 separation system and a 2475 fluorescence detector (Waters, US). HPLC analysis was performed using a Sunfire Prep silica column (4.6 × 250 mm, 5 μm) separation unit, and a mobile phase of n-hexane/tetrahydrofuran (93:7, v/v) with a flow rate of 0.8 ml/min and an injection volume of 10 µl. The temperature of the column was set at 30 °C. The fluorescence detector’s excitation and emission wavelengths were 295 and 330 nm, respectively^24^.

### Statistical analysis

Mean values with standard deviations were used to express the data. SPSS statistical software (version 19) was used to perform the statistical analysis of the data. Analysis of variance followed by Duncan’s multiple range test was used to determine significant differences among the treatments. Also, Statistical significance between treated and untreated samples was calculated using the t-test, and a P-value < 0.05 was considered statistically significant.

## Results and discussion

### The effect of gas type on the aflatoxin concentration and peroxide value

The residual levels of aflatoxin B_1_ in sesame seeds after DBD treatments with different gases are shown in Table 1. Air gas was more effective than argon gas and wet argon in the decomposition of aflatoxin in sesame seeds. In fact, the concentration of aflatoxin under argon gas and wet argon treatment was not significantly different, whereas the concentration of aflatoxin under air gas treatment decreased from 65 ppb to 47 ppb (27.7% reduction). Air has the greatest diversity and intensity of ROS and RNS production. Argon, and more intensely, humid argon, has the greatest concentration of ROS, such as H_2_O_2_^25,26^. Air is a mixture of nitrogen and oxygen. This combination leads to the production of a variety of chemically active species, including atomic oxygen (O), ozone (O₃), hydroxyl radicals (•OH), and reactive nitrogen compounds (RNS). The reduction of aflatoxin when exposed to air gas may be caused by oxygen molecules in the air, which help create reactive oxygen species (like ozone and peroxide) that decompose aflatoxin B_1_ by attacking the double bonds and oxidizing aromatic rings in the aflatoxin structure^27,28^. In addition, as shown in Table 1, the amount of sesame seed peroxide under air gas treatment increased significantly compared with the other two gases. This is probably due to the presence of oxygen and the formation of reactive oxygen species such as ozone and other oxidizing species in the air, which induce oxidation rates and ultimately lead to elevated peroxide levels^29^. Shi et al. (2017) investigated the effect of high-voltage atmospheric cold plasma (HVACP) with gas type (air, MA65), relative humidity (5, 40, 80% RH), and treatment time (1, 2, 5, 10, 20, and 30 min) on the degradation of aflatoxin in corn. The most effective result was observed when HVACP was used for 10 min in MA65 gas at 40% RH, resulting in an 82% of aflatoxin reduction. ​​​​​​​In this study, the reduction in aflatoxin was attributed to the higher concentrations of active ozone and NOx species in the MA65 gas during HVACP treatment compared with air^27^. In addition, Siciliano et al. (2016) evaluated the effect of atmospheric cold plasma produced by different gasses (N_2_, 0.1% O_2_ and 1% O_2_, 21% O_2_) at different times (1, 2, 4, 12 min) and at different powers (1150, 400, 700, 1000 W) on the reduction of aflatoxin B_1_, G_1_, B_2_, G_2_ in hazelnuts. The findings displayed that the greatest aflatoxin detoxification effect (70%) was achieved with nitrogen gas or a nitrogen /oxygen mix (0.1% O2) at the highest exposure time and with a power of 1000 W^6^. According to the observed results, air gas had a significant effect in the reduction of the aflatoxin B_1_ concentration while it increased the peroxide value. Since sesame seeds are vegetable seeds with high oil content and prone to oil oxidation, argon gas, which has less effect on this parameter, was the most suitable gas for the rest of the steps.

**Table 1:** The effect of gas type on the concentration of aflatoxin B_1_ and peroxide value in Sesame seeds.

Sample	Aflatoxin concentration (ppb)	Reduction percentage (%)	Peroxide value (meq/kg (
Control	65 ± 0.41^a^	0	0.61 ± 0.09^d^
Air	47 ± 0.23^c^	27.7	1.6 ± 0.11^a^
Argon	53 ± 0.51^b^	18.47	1.1 ± 0.02^c^
Wet argon	54 ± 0.64^b^	17	1.3 ± 0.02^b^

Data were reported as mean ± SD. Values within each column with different letters are significantly different (*p* < 0.05).

### The effect of cold plasma treatment on aflatoxin concentration

The linear regression equation for the data of this study was y = 626.08x + 113.08, with R^2^ of 0.993. The limit of detection (LOD) was 0.03 µg/kg, and the limit of quantification (LOQ) was 0.1 µg/kg. The results obtained for the second stage are shown in Table 2. The percentage of aflatoxin degradation increased with increasing time and the power of treatment. The highest reduction of AFB_1_ concentration in sesame seeds was observed during 8 min and 56 W, which was a 68.59% destruction rate. In this process, gases such as oxygen and nitrogen are ionized under a strong electric field, producing active radicals such as O•, OH•, O₃, and NO•. These species attack the chemical structure of aflatoxin B_1_ (AFB_1_), specifically targeting the C8 = C9 double bond in the dihydrofuran ring, which is the main cause of the toxicity and carcinogenicity of this toxin. During these reactions, intermediate compounds such as C₁₇H₁₅O₇ are formed, which are much less toxic. Also, the weak ultraviolet light produced in the plasma, along with radicals, causes the molecular bonds to break and the structure of aflatoxin to be destroyed. On the other hand, plasma also inhibits toxin production by damaging the cell membrane of aflatoxin-producing fungi (such as *Aspergillus flavus*)^16^. Overall, as the duration of sesame seed exposure to reactive species in plasma increased, the rate of AFB_1_ degradation also increased. This may be the potential cause of the increase in AFB_1_ degradation after 8 min of DBD treatment^16^. Furthermore, cold plasma can generate a higher number of radicals and charged particles when exposed to high power. Therefore, a power of 56 W was effective for the degradation of AFB_1_ and played an important role in the degradation efficiency^30^. After performing cold plasma treatment, there are two potential ways in which the AFB_1_ can be destroyed: the first includes binding radicals such as CHO., OH., and H. produced by plasma to aflatoxin. The second pathway is peroxidation by HO_2_ radicals and aflatoxin oxidation through the combined effects of oxidative species H_2_O_2_, O_3_, OH. and the reduction of aflatoxin bioactivity due to the loss of the double bond in the furan ring segment, along with the modification of the lactone ring, cyclopentanone and methoxyl group^31^. Iqdiam et al. (2019) investigated the effect of an atmospheric pressure plasma jet (APPJ) to reduce the level of aflatoxin (AFT) in peanuts. ​​​​​​​ The results showed that a significant reduction of aflatoxin after a short period of treatment (after 2 min), AFT decreased from 62.3 ppb to 48.2 ppb (23% decrease) with temperature change (from 24 °C to 92 °C). Also, AFT levels decreased from 64.14 to 39.6 ppb (a reduction of 38%) at in 5 min expoture^32^. Zheng et al. (2023) evaluated the effect of cold plasma dielectric barrier discharge (DBD) on zearalenone degradation in corn. The findings indicated that the rate of zearalenone degradation increased as the treatment time and voltage increased. The level of zearalenone destruction was 56.57% after exposure to 50 kV for 120 s^30^. Moreover, Wielogorsk et al. (2019) investigated the mycotoxin detoxification of corn kernels using cold atmospheric pressure plasma (CAPP) treatment. They indicated that when corn kernels contaminated with AFB_1_ and fumonisin B_1_ were treated using a plasma in 6 kV and 20 kHz and by a mixture of helium and O_2_ gas, the toxin concentration was reduced by 66% in 10 min^33^.

**Table 2:** The effect of cold plasma treatment on the reduction of aflatoxin B_1_ in Sesame seeds.

Sample	Power (W)	Time (min)	Aflatoxin B_1_ concentration (ppb)	Reduction percentage (%)
Untreated	0	0	63.4 ± 037^a^	0
Treated	45	1	37.2 ± 0.25^b^	41.28
		4	35.9 ± 0.46^c^	43.31
		8	32.1 ± 0.15^d^	49.32
	56	1	28.1 ± 0.33^e^	55.61
		4	21.4 ± 0.14^f^	66.21
		8	19.9 ± 0.28^g^	68.59

Data were reported as mean ± SD. Values within each column with different letters are significantly different (*p* < 0.05).

### The effect of cold plasma treatment on the peroxide value

During processing and storage, edible oils are susceptible to oxidation, leading to potential harm to oil quality and human health. It is crucial to measure the peroxide content in edible oils like sesame oil because peroxide levels are a key indicator of lipid oxidation and play a role in maintaining quality standards for edible oils^34^. Because cold plasma is frequently seen as an innovative ionization technology, it is important to examine its impact on the oxidation of oil. As shown in Table 3, there was a significant increase in the amount of peroxide due to the cold plasma treatment. The amount of peroxide in the oil of the control sample was 0.61 ± 0.09 meq/kg, and after plasma treatment with a power of 50 W for 8 min, the peroxide value increased to 1.38 ± 0.12 meq/kg. This may be caused by the generation of free radicals, including reactive oxygen and nitrogen species, excited atoms, ultraviolet rays, and charged particles, during cold plasma processing. These reactive species attack double bonds and produce hydroperoxides, which are formed in the first stage of lipid oxidation. The result of these reactions is an increase in the peroxide value, which indicates the onset of oxidative spoilage in fats^18^. In a similar study, Gebremical et al. (2019) investigated the effect of dielectric barrier discharge plasma with different plasma power (40–10 W), air flow rate (0.5–20 L per minute), and time (15 –1 min) on the amount of peanut peroxide. The findings indicated that as plasma power and time increased and air flow speed decreased, the peroxide values increased. ​​​​​​​They stated that the rise in peroxide levels was related to active species generated during plasma treatment with high oxidation potential, leading to lipid oxidation^34^. Thirumdas et al. (2015) evaluated the amount of peroxide after cold plasma technology on peanuts and walnuts at three input powers (40, 50, and 60 W) and three different times (5, 10, and 15 min). They concluded that the amount of peroxide in the walnut samples increased by 20% at higher powers and times. Similar results were observed for treated peanuts, which may be due to the production of radicals that can oxidize lipid molecules and increase the amount of peroxide^35^.

**Table 3:** The effect of cold plasma treatment on the physicochemical properties of Sesame seeds.

Sample	Peroxide value (meq/kg)	Acid value (mg/g)	PH	L*	b*	a*	Protein (%)
Untreated	0.61 ± 0.09	0.52 ± 0.04	6.88 ± 0.003	44.45 ± 0.35	7.41 ± 0.25	1.21 ± 0.21	19.03 ± 1.00
Treated	1.38 ± 0.12*	0.88 ± 0.02*	6.79 ± 0.005*	42.02 ± 0.21*	9.78 ± 0.15*	2.20 ± 0.16*	18.73 ± 0.95

Results are presented as mean ± SD. T test was performed. *p < 0.05.

### The effect of cold plasma treatment on the acid value

The acid value is influenced by various environmental factors like air, light, moisture, and temperature, and is a measure of the level of free fatty acids in the oils. It is used as an indicator of oil rancidity^36^. Sesame seeds have a high oil content ranging from 44 to 58% and contain high levels of unsaturated fatty acids, which are susceptible to oxidation and rancidity^37^. Table 3 shows the acid values of the control sample and plasma treated at 50 W for 8 min. The oil extracted from the control sesame seeds had an acid value of 0.52 ± 0.04 mg/g, and the oil extracted from the treated sesame seeds had an acid value of 0.88 ± 0.02 mg/g. The increase in acidity during this process may be due to the partial and random hydrolysis of triglyceride molecules to free fatty acids and diacylglycerol^34^. Puprasit et al. (2020) investigated the effect of dielectric barrier discharge (DBD) plasma hydrogenation on the production of margarine. The results showed that after 12–20 h of treatment, the acid value decreased from 0.47 to 0.27%. The decrease in acidity during plasma treatment was due to the generation of free electrons, radicals, and other species in the DBD plasma, which broke down the free fatty acid molecules in palm oil. The result of this study was different from our study. This discrepancy is likely due to differences in the sample matrix, secondary reactions in margarine, and plasma parameters. Sesame seeds contain an oil with high levels of unsaturated fatty acids (such as linoleic acid), which are susceptible to hydrolysis and oxidation. Margarine usually contains higher levels of saturated and hydrogenated fatty acids, which are more stable against plasma reactive species. In margarine, free electrons and radicals produced by the plasma may react with the free fatty acids present, resulting in a decrease in acidity. Also, plasma operating conditions (gas type, contact time, power) can produce different results^36,37^.

### Effect of cold plasma treatment on pH value

pH is one of the most important parameters for evaluating the quality of treated oilseeds. Table 3 shows the pH value of sesame seeds after DBD plasma treatment. ​​​​​​​The pH of the control sample was 6.88 ± 0.003, and a gradual decrease in pH to 6.79 ± 0.005 was observed in the plasma sample. The decrease in pH in the treated samples could be due to the active RNOS species like O^3^O, and NOx interacting with the natural moisture in sesame seeds, resulting in the creation of acid-forming molecules like nitrate and nitrite at very low levels^20^. Similarly, Thirumdas et al. (2016) investigated the effect of cold plasma treatment on the functional and rheological properties of rice starch at power (60 and 40 W). They determined that the pH reduction might result from the formation of acidic compounds such as peroxide groups, formic acid, carbonyl, and carboxyl groups, which are produced through the decomposition of amylose and amylopectin^38^. Aparajhitha et al. (2019) studied the effect of atmospheric pressure cold plasma on the physicochemical properties of Neera coconut. In contrast to our research, this study’s findings suggested that the rise in pH could be attributed to the generation of OH. The increased division of water molecules in Nira is due to the presence of reactive radicals like OH•, and HO_2_, which remove a proton from water, resulting in the generation of additional free radicals. Sucrose in Neera acts as an OH absorber. The hydroxyl radical removes a proton from the sucrose molecule, creating a radical form of sugar lacking a proton. This reaction could explain the increase in pH^39^.

### The effect of cold plasma treatment on color

Color is a significant physical factor that indicates the acceptability of oilseeds by consumers. Food products color is commonly the result of natural or synthetic pigments or chemical reactions. The color of food products may change when cold plasma is used in food processing. Therefore, color plays a significant role in assessing these changes^40^. The color changes of the control sample and those treated with DBD were measured at a power of 56 W and a duration of 8 min, and the results are presented in Table 3. The value of a* (+ redness, -greenness) and the value of b* (+ yellowness, -blue) of the treated seeds increased significantly compared with the control sample. The L* value (+ light, -dark) decreased in treated samples. This indicates that cold plasma treatment increased the number of red, yellow, and dark units. Sesame seeds contain the necessary reactive components (sugars and amine groups in proteins) to generate Maillard reaction products. Therefore, the alteration in color may be linked to the degradation of glycosidic bonds that occurs during plasma treatment, resulting in the production of Maillard reaction browning compounds^41^. Unlike our study, Chaple et al. (2020) evaluated the effect of atmospheric cold plasma on wheat grain and flour color parameters. They noted a reduction in a* and b* values and an increase in L* value as treatment time on wheat grain and flour increased, which was linked to ozone causing damage to the conjugated double bonds of carotenoid pigments^42^. In addition, Lee et al. (2016) in another study reported that the DBD plasma process increased brightness values while reducing yellowness and redness of brown rice, and with increasing treatment time, the whiteness index increased from 56.89 to 58.28 ^22^.

### Effect of cold plasma treatment on protein content

Oilseed protein is crucial for meeting human protein dietary requirements. Therefore, it is necessary to evaluate their content in the treated sesame seed samples^43^. According to Table 3, no significant difference was observed in the protein content of control and treated sesame seeds (56 W and 8 min). The study discovered that dielectric barrier discharge (DBD) plasma treatment commonly impacts the surface of sesame seeds because it does not penetrate deeply; therefore, no significant change in the total protein content was observed^16^. The active components of DBD can impact the protein structure either directly or indirectly through the active species^44^. This result was in accordance with the observations of Lokeswari et al. (2021), who investigated the effect of cold plasma treatment on the protein content of pearl millet. They showed that cold plasma did not have a notable impact on the amount of total protein^45^. In addition, Aparajhitha et al. (2019) also investigated the effect of cold plasma on the amount of protein in Nira coconut and found that plasma treatment did not affect the protein amount^39^. Sarangapani et al. (2017) studied the amount of protein in buckwheat grains effected by cold plasma. In their study, a significant difference was observed in the protein content of the treated and control samples. The protein content of the control buckwheat sample was 23.15%, whereas in the treated samples, it ranged from 23.21 to 23.99% as time and power increased. They stated that the alteration in buckwheat protein levels might result from the direct impact of atomic oxygen and hydroxyl radicals on surface proteins and other protein compounds^21^. In general, cold plasma can cause protein modification and oxidation by generating reactive oxygen species, breaking disulfide bonds and oxidizing thiol groups (-SH), forming carbonyl groups, reducing the solubility and denaturation of proteins, and changing the enzymatic activity or functional properties of proteins. However, in the present study, no changes in protein content were observed, which could be due to the presence of natural antioxidants in sesame seeds or the optimal plasma treatment conditions.

### The effect of cold plasma treatment on texture

Texture is a key factor in determining consumer preference, which is usually associated with sensory attributes. The textural characteristics of sesame seeds, including hardness, fracturability, gumminess, cohesiveness, and springiness, are shown in Table 4. It was clearly observed that DBD plasma affected the textural characteristics of sesame seeds. The hardness of cycles 1 and 2 increased from 335.96 to 277.92 g to 762.45 and 594.68 g, respectively. This result is probably related to the decrease in moisture levels. In addition, the cold plasma contains a high amount of ROS, which oxidizes the free sulfhydryl groups of the protein polypeptide chain that form disulfide bonds, and as a result, creates a strong structure in sesame seeds, which leads to an increase in hardness. Therefore, structural changes in sesame seed protein during plasma treatment may be effective in increasing seed hardness^46^. Miao et al. (2019) reached similar conclusions. They found that with increasing voltage, the hardness of the Alaska Pollock myofibrillar protein increased significantly. They stated that the reason for this phenomenon could be the creation of cross-links and more compact networks in the plasma treatment process^47^. Also, Koddy et al. (2020) investigated the profile of fish muscle tissue under atmospheric cold plasma treatment. They observed that the hardness of the texture increased after 180s compared with the control sample and linked this increase to the oxidation of fish muscle protein and the creation of cross-linked protein networks^48^. Lee et al. (2016) showed that the hardness of brown rice after plasma treatment was significantly lower than that of the untreated sample. They concluded that water absorption and α-amylase activity reduce the hardness of brown rice after plasma treatment, and their results were contrary to the results of our study^22^. In addition, an increasing trend was observed in fracturability, gumminess, and cohesiveness, while the springiness index showed a significant decrease in the treated samples compared with the untreated ones.

**Table 4:** The effect of cold plasma treatment on the textural properties of Sesame seeds.

Sample	Hardness_1_ (g)	Hardness_2_ (g)	Fracturability (g)	Gumminess (g)	Cohesiveness (mJ)	Springiness (mm)
Untreated	335.96 ± 10.11	277.92 ± 14.09	335.96 ± 19.65	36.16 ± 4.13	1.47 ± 0.16	4.14 ± 1.00
Treated	762.45 ± 14.23*	594.68 ± 21.55*	762.45 ± 27.41*	149.82 ± 2.82*	5.06 ± 0.91*	3.45 ± 0.78

Results are presented as mean ± SD. T test was performed. *p < 0.05.

### The effect of cold plasma treatment on phytosterol content

**Fig. 2:**
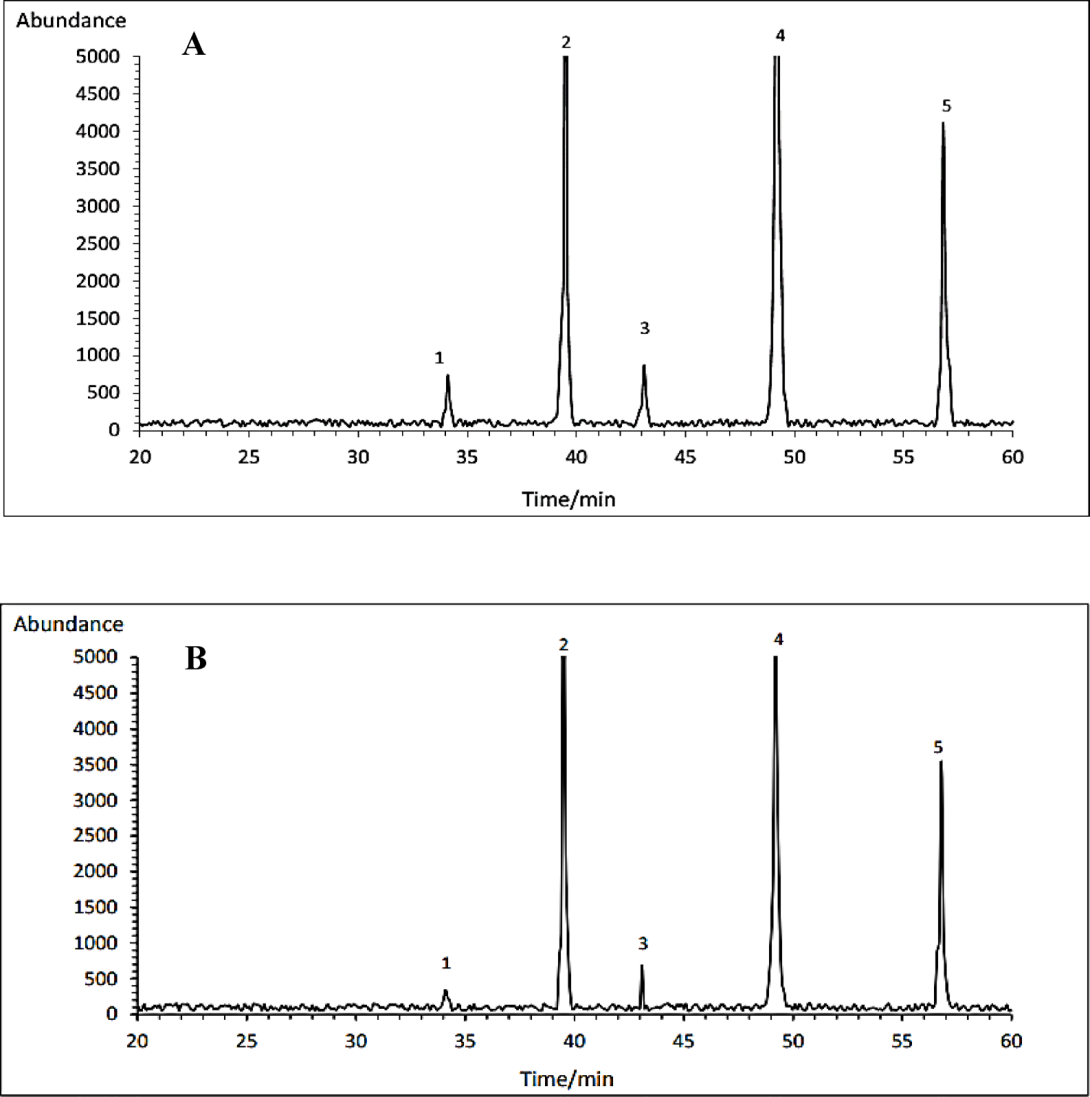
Chromatogram of brassicastrol, campesterol, stigmasterol, β-sitosterol, and Δ7-Avenasterol of sesame oil from left to right for control (**A**) and treated (**B**) samples.

Phytosterols are natural compounds found in the unsaponifiable fat fraction of plant-based foods. Phytosterols are crucial for human health. They can lower LDL cholesterol levels in the bloodstream, which can help prevent cardiovascular diseases, lower the risk of certain cancers, and enhance immune function. According to Fig. 2, the chromatography results showed that the main compounds of phytosterol in sesame oil before and after DBD treatment are brassicasterol, campesterol, stigmasterol, β-Sitosterol, and Δ7-Avenasterol. Plasma treatment had a significant effect on the content of phytosterols; in fact, a significant decrease in the content of brassicasterol from 12.55 to 5.71 mg/g, campesterol from 144.46 to 111.51 mg/g, stigmasterol from 13.04 to 10.19 mg. /g, β-sitosterol from 197.01 to 92.62 mg/g and Δ7-Avenasterol from 73.59 to 63.49 mg/g was observed after plasma treatment. This significant decrease was probably due to the auto-oxidation by active plasma species. Abouelenein et al. (2019) studied the effect of plasma activated water (PAW) at different times (20, 10, 5, and 2 min) on phytosterol content in rocket salad. After PAW treatment, a significant reduction in β-sitosterol and campesterol levels was observed, except for PAW-10, where there was no significant decrease in these compounds^23^. In another study, Yodpitak et al. (2019) analyzed phytochemical changes in six Thai sprouted brown rice varieties under the influence of cold plasma. The findings indicated that one brown rice variety showed a significant reduction in overall phytosterol content after cold plasma treatment, whereas no significant changes were observed in other varieties^49^. Arab et al. (2022) investigated the effect of the roasting process on phytosterol content in white sesame seeds. The results indicated a significant reduction in phytosterol content based on the roasting temperature used. They reported that the reduction in sterol levels may be due to their degradation through hydrolysis, oxidation, or decomposition at high temperatures^50^.

### The effect of cold plasma treatment on antioxidant Lignan levels

**Fig. 3:**
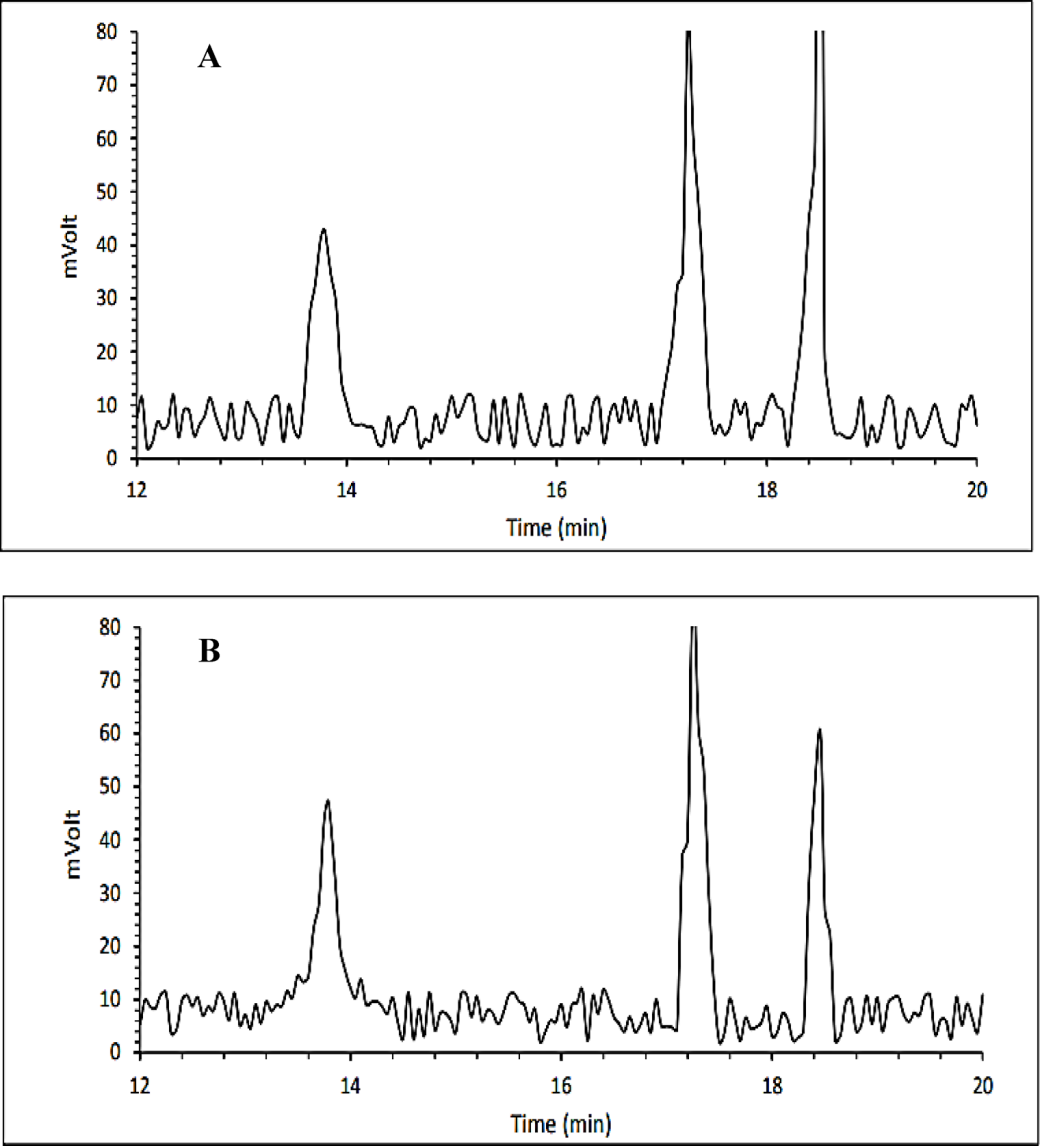
Chromatogram of sesamol, sesamin, and sesamolin of sesame oil, from left to right, respectively, for control (**A**) and treated (**B**) samples.

Sesame seed lignans comprise sesamol, sesamolin, and sesamin. They offer various advantages for enhancing human health, including anti-inflammatory, antioxidant, antidepressant, lipid-lowering, antithrombotic, and cardioprotective effects^50^. Therefore, investigating their changes after plasma treatment is essential. The effect of DBD treatment on the levels of sesamol, sesamin, and sesamolin in sesame oil is shown in Fig. 3. A significant decrease in sesamol content from 70.84 to 34.30 mg/kg, sesamin from 5303.58 to 3946.98 mg/kg, and sesamolin from 2409.3 to 1784.28 mg/kg after treatment was observed. This may be attributed to the contact of plasma reactive oxygen species (peroxyl radicals, hydroxyl radicals, singlet oxygen, and atomic oxygen) with lignins^51^. Geochemical et al. (2019) studied the effect of dielectric barrier discharge plasma on the total polyphenol content of peanuts in different plasma powers (10–40 W), airspeed (0.5–20 L per minute), and time (1–15 min). The results of their study showed that ultraviolet rays and formed oxygen species may lead to an increase in phenolic compounds extracted from cells; therefore, the amount of polyphenols increased after plasma treatment^34^. Makari et al. (2021) assessed the effect of different times (0, 15, 30, 60, 90, 120, 150, and 180 s) of dielectric barrier discharge (DBD) on the total phenol content in pistachio nuts. Their findings indicated that the phenolic content remained unchanged after plasma treatment^51^. Arab et al. (2022) evaluated the effect of the roasting process on the content of sesamin and sesamolin in white sesame seeds for 20 min at different temperatures. When the sesame seeds were roasted at temperatures of 250 and 300 °C, the levels of these two compounds decreased to 344 and 147 mg/100 g, respectively, with a greater decrease in sesamolin than in sesamin. This may be related to the thermal decomposition of sesamolin observed during the roasting process, leading to the formation of other phenolic compounds^50^.

## Conclusions

The results of the first stage with a power of 40 W and a time of 1 min showed that air gas had the greatest decrease in aflatoxin and the greatest increase in the peroxide value. Therefore, in the second stage, the effect of argon gas with powers (45 and 56 W) and different times (1, 4, and 8 min) on the degradation of AFB_1_ and the physicochemical characteristics of sesame seeds was studied. The results showed that DBD treatment reduced the AFB_1_ concentration of sesame seeds by 68.59% in 8 min and at a power of 56 W. The degradation of AFB_1_ was time- and power-dependent. In addition, the physicochemical characteristics of sesame seeds were evaluated under argon gas treatment, 8 min, and 56 W, and compared with the control sample. Effectively, the amount of peroxide, acid value, and textural properties (hardness, fructibility, cohesiveness, gumminess) increased, and pH, springiness index, phytosterol, sesamol, sesamin, and sesamolin decreased. There was no significant difference in protein content. Moreover, plasma treatment darkened the color of the samples treated with DBD compared with the control samples.

Given the non-thermal nature, short processing time, and effectiveness of cold plasma in the laboratory, this technology seems to have high potential for industrial-scale sesame detoxification, but appropriate industrial system design (use of conveyor systems with moving electrodes or fluidized beds to uniformly expose the seeds to plasma), parameter optimization (use of plasma with optimal energy and time), and quality control of the final product (continuous monitoring of sensory and nutritional properties) should be considered.

## Data Availability

The authors declare that the data supporting the findings of this study are available within the article.
